# MF1, a novel robustic acid derivative, exerts antitumor effects in hepatocellular carcinoma

**DOI:** 10.1016/j.tranon.2026.103086

**Published:** 2026-10-26

**Authors:** Juanjuan Huang, Lijuan Teng, Tianxiu Meng, Yuqiong Liang, Yaoqi Li, Jinyan Lv

**Affiliations:** a Faculty of Chinese Medicine Science, Guangxi University of Chinese Medicine, Nanning, Guangxi Zhuang Autonomous Region, 530004, China; b Krirk University, Bangkhen, Bangkok, 10220, Thailand; c School of Public Health, Yale University, New Haven, CT, 06510, USA

**Keywords:** Hepatocellular carcinoma, Antitumor, MF1, Autophagy, Ferroptosis

## Abstract

Hepatocellular carcinoma (HCC) extensive genomic heterogeneity and immunosuppressive tumor immune microenvironment (TIME) are major factors contributing to treatment resistance. This study aims to elucidate the molecular mechanisms by which Robustic Acid 4-O-amino substituted synthetic derivatives (MF1), a novel robustic acid derivative, derived from structural modifications of natural products from *Dalbergia benthamii* Prain, targets carcinogenic signaling and synergistically regulates the crosstalk between autophagy and ferroptosis to induce programmed cell death in HCC. In vitro functional assays confirmed that MF1 exhibits significant antitumor activity. Mechanistically, MF1 significantly upregulates cellular oxidative stress levels, inducing characteristic changes in mitochondrial ultrastructure and apoptosis; simultaneously, by promoting intracellular Fe²⁺ accumulation and downregulating the expression of key ferroptosis regulators GPX4 and SLC7A11, it potently activates the ferroptosis process. Concurrently, MF1 continuously activates the cytotoxic autophagy pathway by regulating the LC3 I/II conversion ratio and upregulating the expression of core autophagy proteins ATG5 and Beclin-1. Network pharmacology identified 7 core targets of MF1 that are common to both ferroptosis and autophagy regulation. qPCR validation showed that MF1 significantly downregulated the mRNA expression of these 7 core oncogenes in HepG2 cells. Molecular docking confirmed strong binding affinities between MF1 and five core targets, 100 ns all-atom molecular dynamics simulations further validated the conformational stability of all five top-ranked MF1-protein complexes, with the higher binding stability for AKT1, SRC and MTOR. In summary, this study clarifies MF1 induces apoptosis and ferroptosis in HepG2 cells, suggesting its potential as a new therapeutic option for HCC.

## Highlights

• MF1, a novel robustic acid derivative, has demonstrated antitumor activity in multiple hepatocellular carcinoma cells.
• MF1 exerts its anticancer effects by simultaneously inducing cytotoxic autophagy and ferroptosis.
• By integrating network pharmacology, molecular docking, and 100-nanosecond all-atom molecular dynamics simulations, AKT1/SRC/MTOR was identified as the core direct targets of MF1.
• Single-cell transcriptomic analysis revealed that AKT1/SRC/mTOR is specifically expressed in tumors and is associated with the immunosuppressive tumor microenvironment in HCC.
• By targeting the interaction between oncogenic signaling and programmed cell death, this approach offers a potential therapeutic strategy for HCC.

## Graphical abstract

**Figure:**
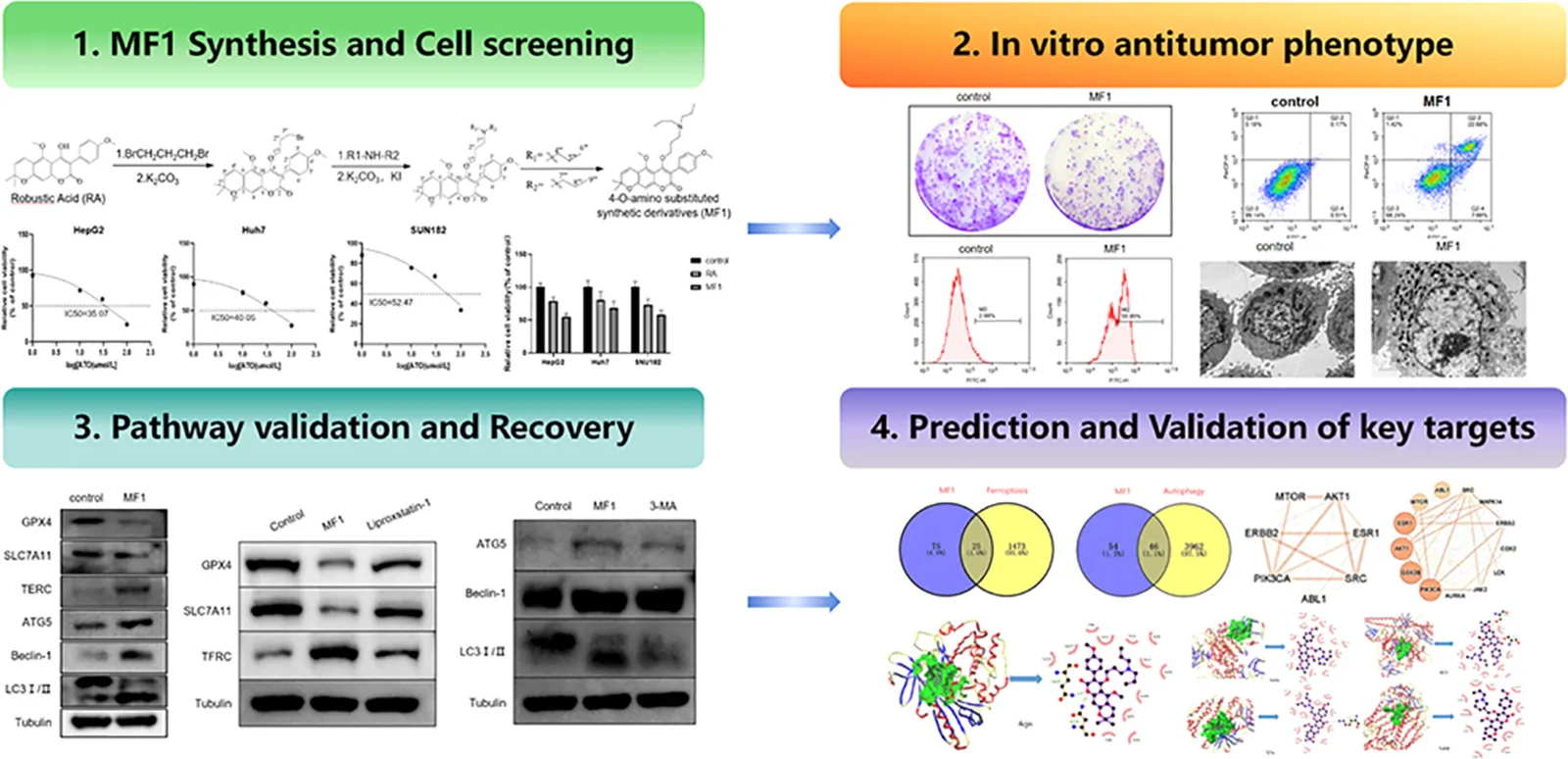

## Introduction

Liver cancer is the sixth most common cancer worldwide and a leading cause of cancer-related deaths^[^1^]^. Hepatocellular carcinoma (HCC) is the primary histological subtype, accounting for approximately 80% of all primary liver cancers^[^2^]^. The prognosis for HCC remains poor due to its high heterogeneity, treatment resistance, and an immunosuppressive tumor immune microenvironment (TIME)^[^3^]^. Oncogenic mutations in HCC are most commonly found in the TERT promoter, TP53, and the Wnt/β-catenin and PI3K/AKT/mTOR pathways^[^4^]^. Targeting these oncogenic mutations and signaling pathways is crucial for developing biomarker-based strategies to overcome treatment resistance in HCC.

Natural product derivatives are an important source of novel antitumor drug candidates^[^5^]^. The strategy of using natural active components as the parent structure and chemically synthesizing a series of derivatives or analogs, from which novel, effective, and low-toxicity active compounds or lead compounds can be screened, is attracting increasing attention to overcome treatment resistance in HCC. Robustic acid (RA), a pyranocoumarin compound with antitumor activity, was isolated and identified from *Dalbergia benthamii* Prain^[^6^]^. RA derivatives have emerged as promising antitumor agents due to their structural versatility, demonstrated by straightforward synthetic accessibility, structural tunability, enhanced solubility, and optimal membrane permeability^[^7^,^^8^^]^. Notably, their inherently conjugated scaffold not only confers exceptional photophysical properties, including intense fluorescence emission and remarkable photostability, but also positions them as ideal fluorophores for advanced probe design in bioimaging applications^[^9^]^. Building upon the structural framework of RA, Robustic Acid 4-O-amino-substituted synthetic derivatives (MF1) were synthesized through synthetic methodologies including nucleophilic substitution and click chemistry in our preliminary studies^[^10^,^^11^^]^.

Ferroptosis is an iron-dependent form of cell death that is typically accompanied by lipid peroxidation and ultimately results in regulated cell death^[^12^,^^13^^]^. Recent studies have demonstrated the critical role of ferroptosis in regulating tumor growth, including HCC^[^14^,^^15^^]^, which provides new insights for the treatment. Furthermore, polymorphisms in the CISD1 and TP53 genes (S47 variants) have been demonstrated to negatively regulate ferroptosis^[^16^,^^17^^]^. RB, NRF2, and MT1G have been shown to protect HCC from sorafenib-induced ferroptosis^[^[18], [19], [20]^]^. This evidence suggests that drugs targeting ferroptosis may be a potential therapeutic option for HCC.

Autophagy is a cellular self-degradation process that maintains cellular homeostasis by engulfing damaged proteins and organelles into autophagosomes, which are then transported to lysosomes for degradation^[^21^,^^22^^]^. Recent studies have shown that upregulation of autophagy-related genes ATG5 and ATG7 can promote the survival and progression of HCC cells^[^23^]^. Autophagy inhibitors such as chloroquine and 3-MA can enhance the therapeutic effects of Sorafenib on HCC by inhibiting autophagy to suppress tumor cell growth^[^24^,^^25^^]^. These studies suggest that drugs targeting the autophagy pathway could become a potential therapeutic strategy for HCC.

In this study, we investigated the antitumor activity of MF1 in HCC cells, and explored the mechanism by which MF1 targets HCC-associated oncogenic pathways to regulate the crosstalk of autophagy and ferroptosis. The findings of this study offer a more accurate prognostic assessment and potential new therapeutic approaches for HCC.

## Materials and methods

### Cell and mice

Human HCC cell lines (HepG2, Huh7, SNU182) were obtained from Guangzhou Shuangquan Biotechnology Co., Ltd. Cells were cultured in Dulbecco's Modified Eagle Medium (DMEM) supplemented with 10% fetal bovine serum (FBS), 1% penicillin-streptomycin, and maintained in a humidified incubator with 5% CO₂ at 37 °C. For functional assays, cells were divided into a vehicle control group and MF1 treatment group. The MF1 group was treated with 35.07 μM MF1 for 48 h, unless otherwise specified. For pathway inhibition studies, cells were pre-treated with the following inhibitors: autophagy inhibitor 3-Methyladenine (3-MA, 5 mM, Sigma-Aldrich), ferroptosis inhibitor Liproxstatin-1 (Lip-1, 15 µM, MedChemExpress).

8 weeks old BALB/c nude mice were purchased from the Central Laboratory of Animal Science of Guangxi University of Chinese Medicine. All mice were housed in a specific-pathogen-free facility, with unrestricted access to autoclaved water and food. All animal-related procedure conformed to the U.S. Public Health Service Policy on Humane Care and Use of Laboratory Animals and were reviewed and approved by the Ethics Committee on Use and Care of Animals of Guangxi University of Chinese Medicine.

### Cell viability detection

HepG2, Huh7, and SNU182 cells were seeded into 96-well plates and allowed to adhere overnight. Cells were then treated with gradient concentrations of MF1 for 48 h. Cell viability was quantified using the Cell Counting Kit-8 (CCK-8) assay according to the manufacturer's instructions, and half-maximal inhibitory concentration (IC₅₀) values were calculated via dose-response curve modeling.

### Colony formation assay

HepG2 cells were seeded into 6-well plates and allowed to adhere overnight. Cells were treated with 35.07 μM MF1, and the medium was refreshed every 3 days. The experiment was terminated when visible cell colonies formed (approximately 14 days). Cells were washed twice with 1 × PBS, fixed with anhydrous methanol for 20 min, stained with 0.5% crystal violet solution for 15 min, rinsed thoroughly with running water, and air-dried. Colonies were imaged and counted for statistical analysis.

### Biochemical detection of MDA, GSH/GSSG, and ferrous ions

The levels of malondialdehyde (MDA), glutathione (GSH)/oxidized glutathione (GSSG), and ferrous (Fe²⁺)/ferric (Fe³⁺) ions in HepG2 cell pellets were measured using commercial detection kits, following the manufacturer's protocols. Optical density (OD) values were measured using a microplate reader, and relative concentrations were calculated against standard curves.

### Apoptosis quantification by flow cytometry

HepG2 cell apoptosis was quantified using the Annexin V-FITC/PI Apoptosis Assay Kit, following the manufacturer's staining protocol. After treatment, cells were harvested, washed with PBS, and incubated with Annexin V-FITC/PI staining solution for 15 min at room temperature in the dark. Apoptosis rates were analyzed using a flow cytometer (Beckman, MoFlo Astrios EQ).

### ROS detection

Intracellular reactive oxygen species (ROS) production was quantified using a ROS detection kit with the DCFH-DA fluorescent probe, following the manufacturer's instructions. After treatment, cells were incubated with DCFH-DA for 20 min at 37 °C, washed with serum-free medium, and intracellular ROS levels were analyzed via flow cytometry.

### Western blot

HepG2 cells were lysed in RIPA lysis buffer supplemented with 1% PMSF. Lysates were centrifuged at 12,000 rpm for 10 min at 4 °C, and the supernatant was collected. Total protein concentration was determined using a BCA Protein Assay Kit (cat# BCA02, Shanghai Dingguo Biotechnology). Equal amounts of protein were denatured by boiling, separated via 10% SDS-PAGE, and transferred to PVDF membranes. Membranes were blocked with 5% non-fat milk in PBST for 1 h at room temperature, then incubated with primary antibodies overnight at 4 °C. After washing with PBST, membranes were incubated with appropriate horseradish peroxidase-conjugated secondary antibodies for 1 h at room temperature. Protein bands were visualized using a hypersensitive chemiluminescent reagent (Biosharp, cat #BL520A) and a BIO-RAD Gel Doc XR+ imaging system. Band density was quantified using ImageJ software (NIH, USA).

### Quantitative real-time PCR (qPCR)

Total RNA was extracted from HepG2 cells using a total RNA extraction reagent (BL258A, Biosharp, China) via the chloroform/isopropanol method. RNA was reverse-transcribed into cDNA using the PrimeScript™ RT Master Mix (RR036A, TAKARA, Japan). qPCR was performed using 2×UniTaq SYBR qPCR Master Mix (ROX-) (P1504, TIANYAbiotech, Guangzhou) to detect the mRNA expression of target genes. Primers for target genes were designed and synthesized by Guangzhou All-Perfect Biological Technology Co., Ltd. (primer sequences in Table 1).

**Table 1:** The sequences of primers.

**Gene name**	**sequences**
AKT1	F:5′-GCAGGATGTGGACCAACGTGAG-3′
	R:5′-CAGGCAGCGGATGATGAAGGTG-3′
ESR1	F:5′-GGTCAGTGCCTTGTTGGATGCT-3′
	R:5′-CTGCCAGGTTGGTCAGTAAGCC-3′
ABL1	F:5′-GGGCGTGTGGAAGAAATACA-3′
	R:5′-TGGATAATGGAGCGTGGTGA-3′
GAPDH	F:5′-TGCCACTCAGAAGACTGTGG-3′
	R:5′-TTCAGCTCTGGGATGACCTT-3′
ERBB2	F:5′-TTGAGTCCATGCCCAATCCC-3′
	R:5′-TTGTCCTCAAAGAGCTGGGT-3′
PIK3CA	F:5′-GCAAGTCGAGGCAATGGAAA-3′
	R:5′-ACAGTCCATTGGCAGTTGAG-3′
SRC	F:5′-TGCCCAGCTATGAGAGACAG-3′
	R:5′-CTGAGGCTAGAGGGGAAAGG-3′
MTOR	F:5′-AAAGGCCATGAAGATCTGCG-3′
	R:5′-TACTGCAGCACTTTGGGGAT-3′

### Electron microscope photography

HepG2 cells were harvested via trypsinization, washed twice with PBS, and fixed with 2.5% glutaraldehyde followed by 1% OsO₄ for 2 h. Samples were subjected to gradient dehydration with ethanol (15 min per gradient, 3 × 100% ethanol), followed by acetone replacement (3×). Samples were then impregnated with a mixture of embedding agent and acetone for 4 h, incubated in pure embedding agent overnight, and polymerized at 60–70 °C overnight. Ultrathin sections were prepared using a LEICA EM UC7 ultramicrotome, stained with uranyl acetate and lead citrate, and imaged via transmission electron microscopy.

### Network pharmacology prediction of MF1 targets

First, the SMILES notation of MF1 was entered into the SwissTargetPrediction database to retrieve potential targets associated with MF1. These targets were then validated using the UniProt database. To identify ferroptosis- and autophagy-related targets, we searched the Genecards and PharmGKB databases using the keywords “Ferroptosis” and “Autophagy”, respectively. The ferroptosis and autophagy-related targets were then imported into the Venny 2.1.0 database to identify the intersection of the targets associated with the synthetic derivative and ferroptosis, as well as the intersection of synthetic derivative targets and autophagy-related targets. Then, the resulting two sets of intersecting targets were imported into the STRING database to construct a protein-protein interaction (PPI) network. The network data was saved in CSV format and analyzed using Cytoscape 3.9.1 for topological analysis. Core targets were selected based on Betweenness, Closeness, and Degree values greater than or equal to the average values. These core targets are highly likely to be the key targets through which the synthetic derivative exerts its effects on ferroptosis and autophagy.

### Molecular docking validation

The 3D crystal structures of core target proteins were downloaded from the RCSB Protein Data Bank. Molecular docking simulations between MF1 and core targets were performed using Sybyl-X 2.0 software, with the Total Score used to evaluate binding affinity. Ligand-receptor interactions were visualized and analyzed using Ligplot software to characterize the binding mode of MF1 to its core targets.

### Molecular dynamics simulation

Molecular dynamics (MD) simulations of protein-ligand complexes were performed using the GROMACS 2025.3 software package^[^26^]^, with the AMBER14SB force field and the TIP3P water model for solvation. The atomic partial charges and optimized molecular geometries of the ligands were obtained through quantum mechanical (QM) calculations using the ORCA program^[^27^]^. Small-molecule topology files were generated using the sobtop software, and ions were added to physiological concentration (150 mM NaCl). Perform a 500-step energy minimization using the conjugate gradient method (convergence tolerance 100.0 kJ/mol·nm, step size 0.01 nm), followed by a 100 ps (time step 1 fs) constrained dynamics simulation and a 100 ns (time step 2 fs) formal simulation. Temperature was maintained at 298.15 K using the V-rescale method (time constant 0.2 ps), and pressure was maintained at 1.0 bar using the C-rescale method (time constants 0.5 ps and 2.0 ps). LINCS was used to constrain hydrogen bonds, PME was used to handle long-range electrostatic interactions (cutoff radius 1.0 nm), and the Verlet truncation method was used to handle van der Waals interactions (cutoff radius 1.0 nm), with energy and pressure dispersion corrections enabled. System center-of-mass motion was removed during the simulation, and angular momentum removal with independent temperature control coupling was applied to the protein-ligand complex during the main simulation phase. Analyses included: root-mean-square deviation (RMSD) and gyration radius (Rg) of protein main-chain Cα atoms, root-mean-square fluctuation (RMSF) of residues, solvent-accessible surface area (SASA), and the number of hydrogen bonds between the protein and MF1.

### Single-cell and immune infiltration analysis

Single-cell transcriptomic data from the LIHC_GSE166635 dataset were loaded from the TISCH2 (Tumor Immune Single-Cell Hub 2) database, and expression data for the target genes (AKT1, SRC, MTOR) were extracted. UMAP (Uniform Manifold Approximation and Projection) was used for dimensionality reduction, and the figure shows the distribution of the single-cell data after UMAP reduction. Each point in the figure represents a single cell, and the position of the point reflects the similarity of the cell’s expression profile in the high-dimensional space. Through UMAP dimension reduction, different cell types are clearly separated based on their unique expression patterns. Cells were color-coded based on cell type or the expression levels of specific genes. For AKT1, SRC, and MTOR, the AUCell algorithm was used to assign gene set activity scores to each cell; this algorithm is based on the AUC (Area Under the Recovery Curve) statistic for gene rankings. After scoring, the following visualizations were generated: a Nebulosa kernel density estimate plot showing regions of high activity; a multi-gene dot plot (Seurat DotPlot), where color maps the average expression of each gene across cell types and bubble size maps the expression proportion, displayed separately for MainLabel and FineLabel. The expression of AKT1, SRC, and MTOR is paired with immune cell infiltration scores from TIMER 2.0 on a sample-by-sample basis. ESTIMATE algorithm was used to calculate stromal, immune, and ESTIMATE scores for each sample, and correlations with target gene expression were analyzed. For each gene and each immune cell infiltration indicator, the Spearman correlation coefficient is calculated to assess the strength of the relationship. The Benjamini-Hochberg method is used for multiple comparison correction across all gene-immune cell correlations, and both raw P-values and corrected results are output for comprehensive analysis. Visualizations are implemented using the ggplot2 R package.

### Statistical analysis

Statistical analyses were performed using GraphPad Prism 9.0. Differences between two groups were evaluated using Student's *t*-test, while differences among multiple groups were analyzed using one-way ANOVA followed by Bonferroni post-hoc tests. Data are presented as mean ± standard error of the mean (SEM). A p-value < 0.05 was considered statistically significant. All experiments were independently replicated at least three times.

## Results

### MF1 exerts potent cytotoxicity in HCC cells via autophagy and ferroptosis pathways

RA has been shown to exert antitumor activity in multiple cancer types, and we synthesized a series of RA analogs including MF1 to improve antitumor potency (Fig. 1A). ¹³C NMR and MS results confirmed the purity of MF1 was 96.2% (Fig. 1B**–**C). We first evaluated the cytotoxicity of MF1 in three human HCC cell lines (HepG2, Huh7, SNU182), with IC₅₀ values of 35.07 μM, 40.05 μM, and 52.47 μM at 48 h for HepG2, Huh7, and SNU182 cells, respectively (Fig. 1D). Add RA or MF1 increases anti-tumor activity via CCK-8 assay (Fig. 1E). HepG2 cells, which showed the highest sensitivity to MF1, were selected for subsequent mechanistic studies. Colony formation assays showed that MF1 treatment significantly reduced the number of viable colonies in HepG2 cells compared to the vehicle control group (P < 0.001) (Fig. 1F). Flow cytometric analysis revealed that MF1 treatment induced increase in total apoptotic cells compared to the vehicle control group (P < 0.01) (Fig. 1G). These results confirm that MF1 exhibits significant anticancer effects in HCC.

**Fig. 1:**
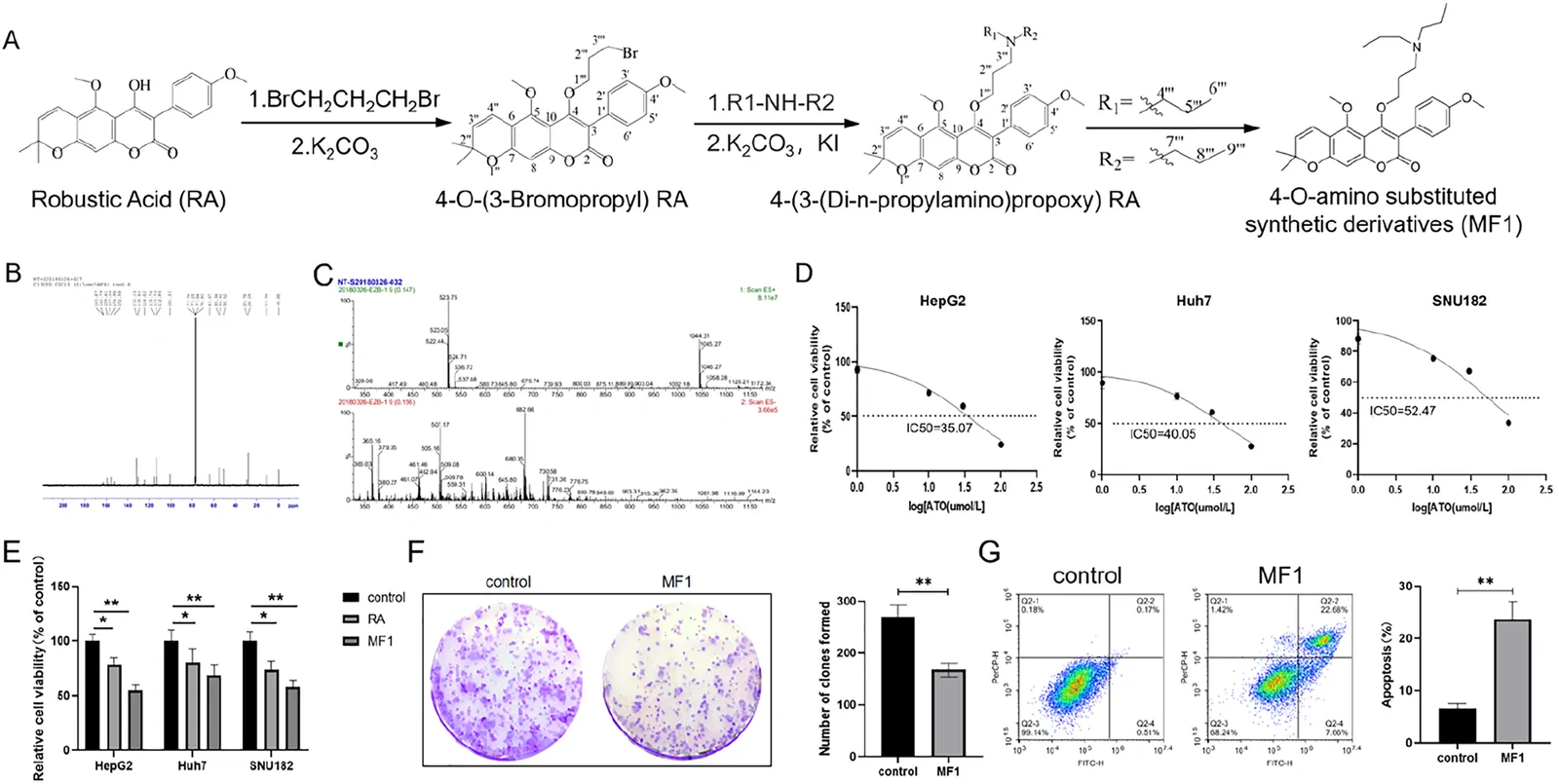
**MF1 exerts potent cytotox. A.** Chemical structures of RA and its derivative MF1. **B-C.** Purity validation of MF1 by ¹³C NMR and MS. **D.** IC₅₀ values of MF1 in HepG2, Huh7, and SNU182 cells at 48 h. **E.** Cell viability of HepG2, Huh7, and SNU182 cells treated with control, RA, or MF1. CCK8 detects the IC50 of MF1 for HepG2. **F.** Colony formation assay showing the inhibitory effect of MF1 on HepG2 clonogenicity. **G.** Flow cytometric analysis of apoptosis in control and MF1-treated HepG2 cells. Data are shown as means ± s.e.m. of three independent experiments. Significant difference between the groups, *P < 0.05; **P < 0.01.

### MF1 induces autophagy and ferroptosis in HepG2 cells

We next evaluated the effects of MF1 on oxidative stress, a core upstream mediator of both autophagy and ferroptosis. MF1 treatment induced a significant increase in intracellular ROS levels (P < 0.001) and MDA content (P < 0.01) in HepG2 cells (Fig. 2A**–**B). Transmission electron microscopy revealed marked ultrastructural changes in MF1-treated HepG2 cells, including mitochondrial contraction (a characteristic morphological feature of ferroptosis) and the formation of autolysosomes (a hallmark of autophagy activation) (Fig. 2C). We further quantified key markers of ferroptosis and redox homeostasis. MF1 treatment induced a significant increase in relative Fe²⁺ content (P < 0.001) and a concomitant decrease in relative Fe³⁺ content (P < 0.001) in HepG2 cells (Fig. 2D). Additionally, MF1 treatment significantly reduced intracellular GSH levels (P < 0.001) and increased GSSG levels (P < 0.001), resulting in a marked reduction in the GSH/GSSG ratio, a key indicator of ferroptosis activation (Fig. 2E). Western blot analysis confirmed the activation of both ferroptosis and autophagy pathways at the protein level (Fig. 2F). For ferroptosis-related proteins, MF1 treatment significantly downregulated the expression of GPX4 and SLC7A11, and upregulated the expression of TFRC. For autophagy-related proteins, MF1 treatment significantly increase the conversion of LC3-I to LC3-II, resulting in a decreased LC3-I/LC3-II ratio, and upregulated the expression of ATG5 and Beclin-1. Collectively, these results demonstrate that MF1 simultaneously induces autophagy and ferroptosis in HepG2 cells.

**Fig. 2:**
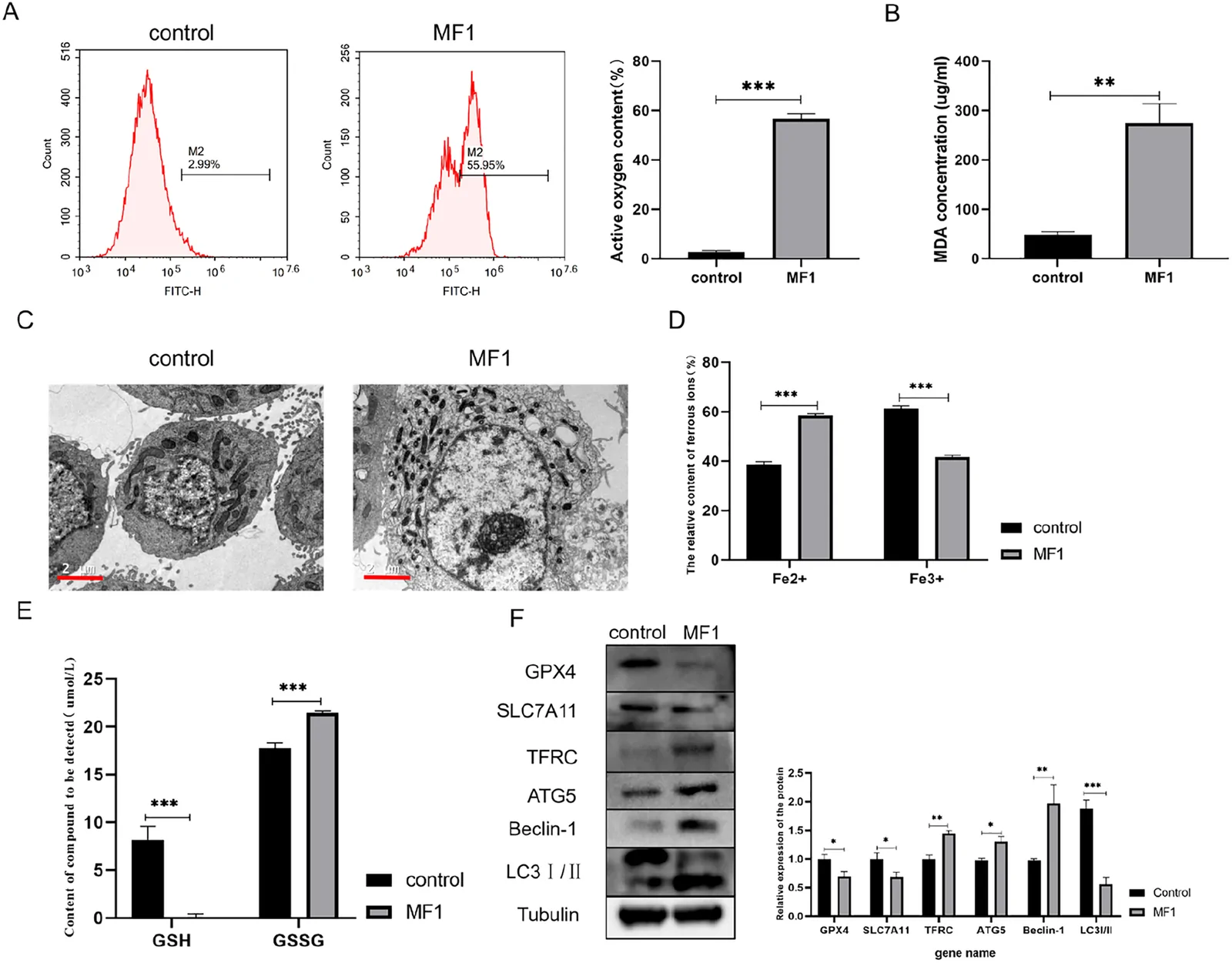
**MF1 induces autophagy and ferroptosis in HepG2 cells. A.** ROS levels in control and MF1-treated HepG2 cells. **B.** MDA content in HepG2 cells after MF1 treatment. **C.** TEM images showing mitochondrial shrinkage and autophagic vacuoles induced by MF1 (scale bar: 2 μm). **D.** Relative levels of Fe²⁺ and Fe³⁺ in HepG2 cells. **E.** Intracellular GSH and GSSG levels. **F.** Western blot and quantitative analysis of ferroptosis-related proteins (GPX4, SLC7A11, TFRC) and autophagy-related proteins (ATG5, Beclin-1, LC3 I/II). Data are shown as means ± s.e.m. of three independent experiments. Significant difference between the groups, *P < 0.05, **P < 0.01, ***P < 0.001.

### The inhibitor of autophagy and ferroptosis blocks MF1-induced antitumor effects in HepG2 cells

To further clarify the critical roles of autophagy and ferroptosis in the antitumor effects of MF1, we performed functional rescue experiments using the selective autophagy inhibitor 3-MA and ferroptosis inhibitor Lip-1. CCK-8 assays showed that pre-treatment with either Lip-1 (P < 0.05) or 3-MA (P < 0.001) significantly increased the viability of HepG2 cells compared to the MF1 alone group (Fig. 3A). Flow cytometric analysis of apoptosis further validated these findings: pre-treatment with Lip-1 significantly reduced the apoptosis rate of MF1-treated HepG2 cells (P < 0.05), while pre-treatment with 3-MA induced an even more pronounced reduction in apoptosis rate (P < 0.001) (Fig. 3B). We also found that inhibition of either pathway significantly reversed MF1-induced changes in key functional markers. Both Lip-1 and 3-MA pre-treatment significantly reduced intracellular ROS and MDA levels (Fig. 3C**–**D), restored GSH/GSSG homeostasis (Fig. 3E), decreased Fe²⁺ accumulation (Fig. 3F), and reversed the mitochondrial contraction and autolysosome formation induced by MF1 in HepG2 cells decreased Fe²⁺ accumulation (Fig. 3G). Western blot analysis confirmed that Lip-1 pre-treatment significantly restored the expression of ferroptosis-related proteins and 3-MA reversed the molecular changes induced by MF1(Fig. 3H**–**I). These results confirm that both autophagy and ferroptosis are essential mediators of the antitumor effects of MF1 in HCC cells.

**Fig. 3:**
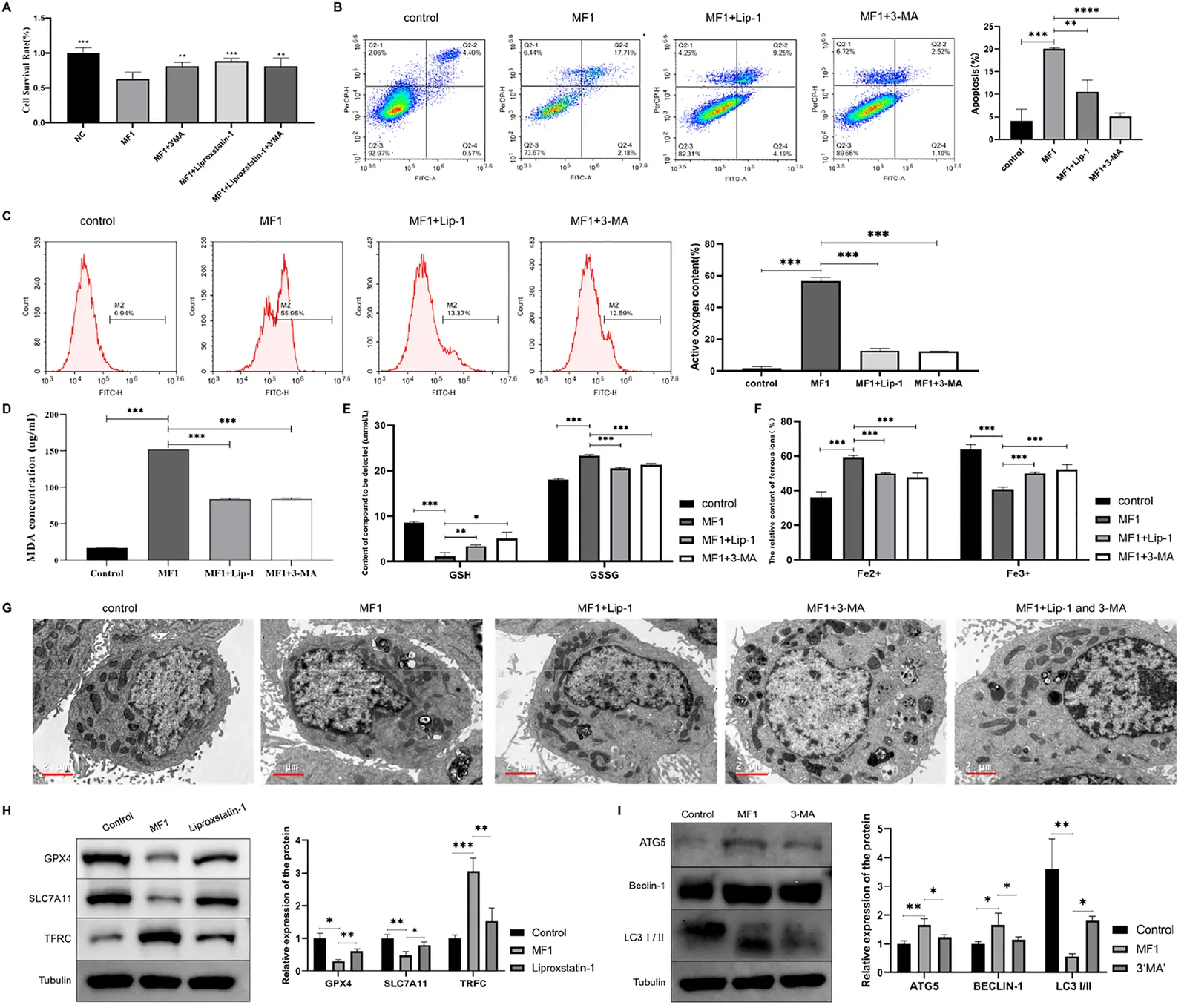
**The inhibitor of autophagy and ferroptosis blocks MF1-induced antitumor effects in HepG2 cells. A.** Cell viability of HepG2 cells in control, MF1, MF1 with Liproxstatin-1, MF1 with 3-MA, and MF1 with Liproxstatin-1 + 3-MA groups. **B.** Flow cytometric analysis of apoptosis in the four groups. **C.** Intracellular ROS levels. **D-E.** MDA content and GSH/GSSG ratio. **F.** Relative levels of Fe²⁺ and Fe³⁺. **G.** TEM images showing morphological recovery after Liproxstatin-1, 3-MA and Liproxstatin-1 + 3-MA pretreatment (scale bar: 2 μm). **H-I.** Western blot and quantitative analysis of ferroptosis and autophagy marker proteins. Data are shown as means ± s.e.m. of three independent experiments. Significant difference between the groups, *P < 0.05, **P < 0.01, ***P < 0.001.

### MF1 targets core oncogenic drivers in HCC

To decode the upstream molecular mechanism by which MF1 regulates autophagy and ferroptosis, we performed integrated network pharmacology analysis. A total of 100 potential targets of MF1 were identified via the Swiss Target Prediction database. We further retrieved 1498 ferroptosis-related genes, 4008 autophagy-related genes by Genecards and PharmGKB databases. Venny intersection analysis identified 25 overlapping targets between MF1 and ferroptosis, 45 overlapping targets between MF1 and autophagy, and 7 core targets common to both pathways that are also established HCC driver genes regulators: ABL1, ERBB2, PIK3CA, SRC, ESR1, mTOR, and AKT1(Fig. 4A, Table 2). PPI network and topological analysis confirmed that these 7 core genes are key hub nodes linking oncogenic signaling, autophagy and ferroptosis regulation in HCC (Fig. 4B). qPCR validation showed that MF1 treatment significantly downregulated the mRNA expression of all 7 core genes in HepG2 cells (P < 0.05) (Fig. 4C), confirming that MF1 directly modulates the expression of these HCC oncogenic drivers at the transcriptional level. Molecular docking simulations further confirmed the direct binding of MF1 to these core target proteins (Fig. 4D, Table 3). The majority of the core targets exhibited strong binding affinities with MF1, with the highest Total Score for binding to AKT1 (10.6673), followed by SRC (10.1721), JAK2 (9.8996), mTOR (9.5120), and ERBB2 (9.0998). Ligplot visualization revealed that MF1 binds stably to the active pockets of these target proteins via hydrogen bonds and hydrophobic interactions: MF1 inserts into the active cavity of AKT1, forming hydrogen bonds with Gln79 and Asn54, and hydrophobic interactions with 14 amino acid residues including Leu210, Asp274, and Trp80. MF1 interacts with the active pocket of SRC via extensive hydrophobic contacts with 16 amino acid residues including Gly277, Tyr343, and Asp351. MF1 binds to the active site of mTOR, forming hydrogen bonds with Ser773 and Ser774, and hydrophobic interactions with 13 amino acid residues including Met772, Asp933, and Trp780. MF1 inserts into the active cavity of ERBB2, forming hydrophobic interactions with 13 amino acid residues including Trp266, Leu213, and Phe270. MF1 binds to the active cavity of JAK2, forming a hydrogen bond with Lys667, and hydrophobic interactions with 14 amino acid residues including Val610, Phe631, and Leu579.

**Fig. 4:**
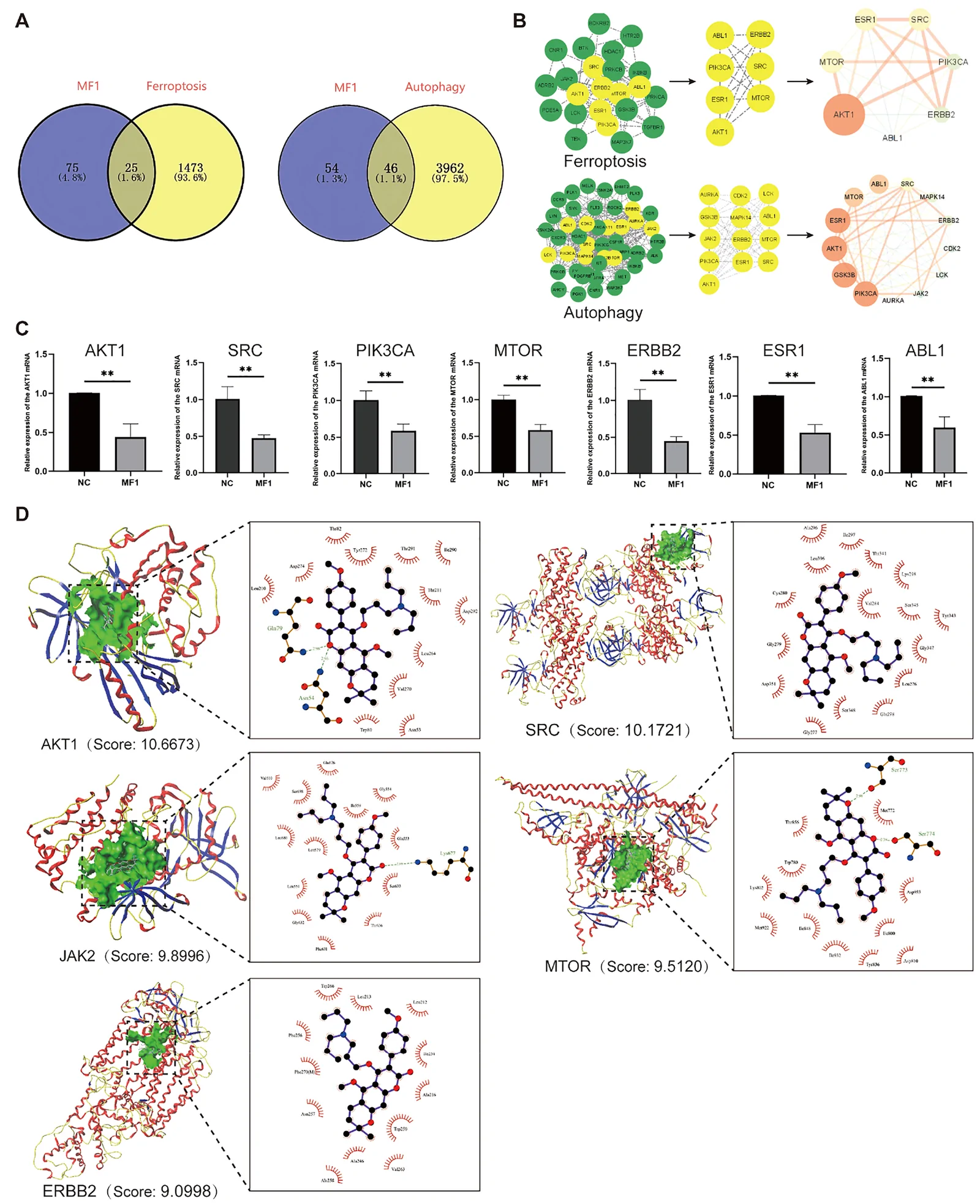
**MF1 Targets Core Oncogenic Drivers in HCC That Orchestrate Mutational Landscape. A.** Venn diagram showing overlapping targets of MF1 with ferroptosis and autophagy. **B.** PPI network of MF1 targets involved in ferroptosis and autophagy. **C.** mRNA expression of seven core hub genes (ABL1, ERBB2, PIK3CA, SRC, ESR1, mTOR, AKT1) after MF1 treatment. **D.** Molecular docking binding patterns of MF1 with AKT1, SRC, mTOR, ERBB2, and JAK2. Data are shown as means ± s.e.m. of three independent experiments. Significant difference between the groups , *P < 0.05, **P < 0.01.

**Table 2:** Corresponding Targets for the Intersection of Two Groups of Targets.

**Keywords**	**Intersection targets**
ferroptosis	HTR2B, ERBB2, ESR1, BDKRB2, IKBKB, SRC, CNR1, PDE5A, TGFBR1, ADRB2, LCK, JAK2, TEK, BTK, MAP3K7, P2RX7, HDAC1, AKT1, GSK3B, AHCY, MTOR, PRKCA, PRKCB, PIK3CA, ABL1
autophagy	PIK3CD, HTR2B, MELK, MET, ERBB2, ESR1, ROCK2, KIT, FLT3, IKBKB, SRC, PDGFRB, CDK2, PLK1, AURKA, PLK3, CNR1, MAPK14, CSNK2A1, CSNK2A2, TGFBR1, EHMT2, ADRB2, PARP1, LCK, KDR, JAK2, LYN, MAP3K7, CXCR3, HDAC1, AKT1, SYK, AHCY, PGK1, CSF1R, GSK3B, CCR5, MTOR, PRKCA, PRKCB, PIK3CA, ABL1, PDGFRA, FYN

**Table 3:** Molecular docking Total Score.

**Target name**	**PDB ID**	**Total Score**
AKT1 SRC JAK2 MTOR ERBB2 ESR1 CDK2 PIK3CA GSK3B LCK AURKA MAPK14	4ejn 6e6e 7f7w 4l23 5o64 6psj 6q4h 5dxh 4afj 1 × 27 3w2c 4lop	10.6673 10.1721 9.8996 9.512 9.0998 8.8627 8.5728 8.4497 8.3598 8.0316 7.0362 7.0029

### Molecular dynamics simulations confirm the stable binding of MF1 to core oncogenic targets

Among the 7 core targets identified by network pharmacology, 5 targets (AKT1, SRC, mTOR, ERBB2, JAK2) with docking scores > 9.0 were selected for molecular dynamics simulation, we performed 100 ns all-atom molecular dynamics simulations in explicit solvent. As shown in Fig. 5A, all five complexes reached conformational equilibrium within the first 20 ns of simulation, and their backbone Root mean square deviation (RMSD) values remained stable thereafter with fluctuations ranging from 0.12 to 0.28 nm. Notably, the AKT1-MF1 complex exhibited the smallest RMSD fluctuation, indicating the highest conformational stability, which is consistent with its highest molecular docking score. Root mean square fluctuation (RMSF) analysis revealed that the fluctuations of residues at key active sites and loop regions of each protein are significantly reduced after combining with MF1(Fig. 5B). This indicates that MF1 binding effectively stabilizes the active site conformations of its target proteins. Solvent accessible surface area (SASA) and radius of gyration (Rg) analyses further confirmed the structural integrity of the complexes. The SASA values of all complexes remained stable throughout the simulation (Fig. 5C), with no significant expansion observed, indicating that MF1 binding did not induce global protein unfolding. Similarly, the Rg values (Fig. 5D) fluctuated within a narrow range (2.1–2.4 nm), demonstrating that the proteins maintained their compact, functional conformations during the simulation. Hydrogen bond analysis showed that MF1 formed stable hydrogen bond interactions with AKT1, SRC and MTOR (Fig. 5E). The AKT1-MF1 complex had the highest average number of hydrogen bonds. These stable hydrogen bond interactions are the primary contributors to the high binding affinity of MF1 for its targets. Free energy landscape analysis revealed MF1 induced a stable binding conformation state, with no significant conformational change occurring (Figs. 5F**–**G). These results indicate that MF1 forms stable complexes with five proteins, especially AKT, SRC and MTOR.

**Fig. 5:**
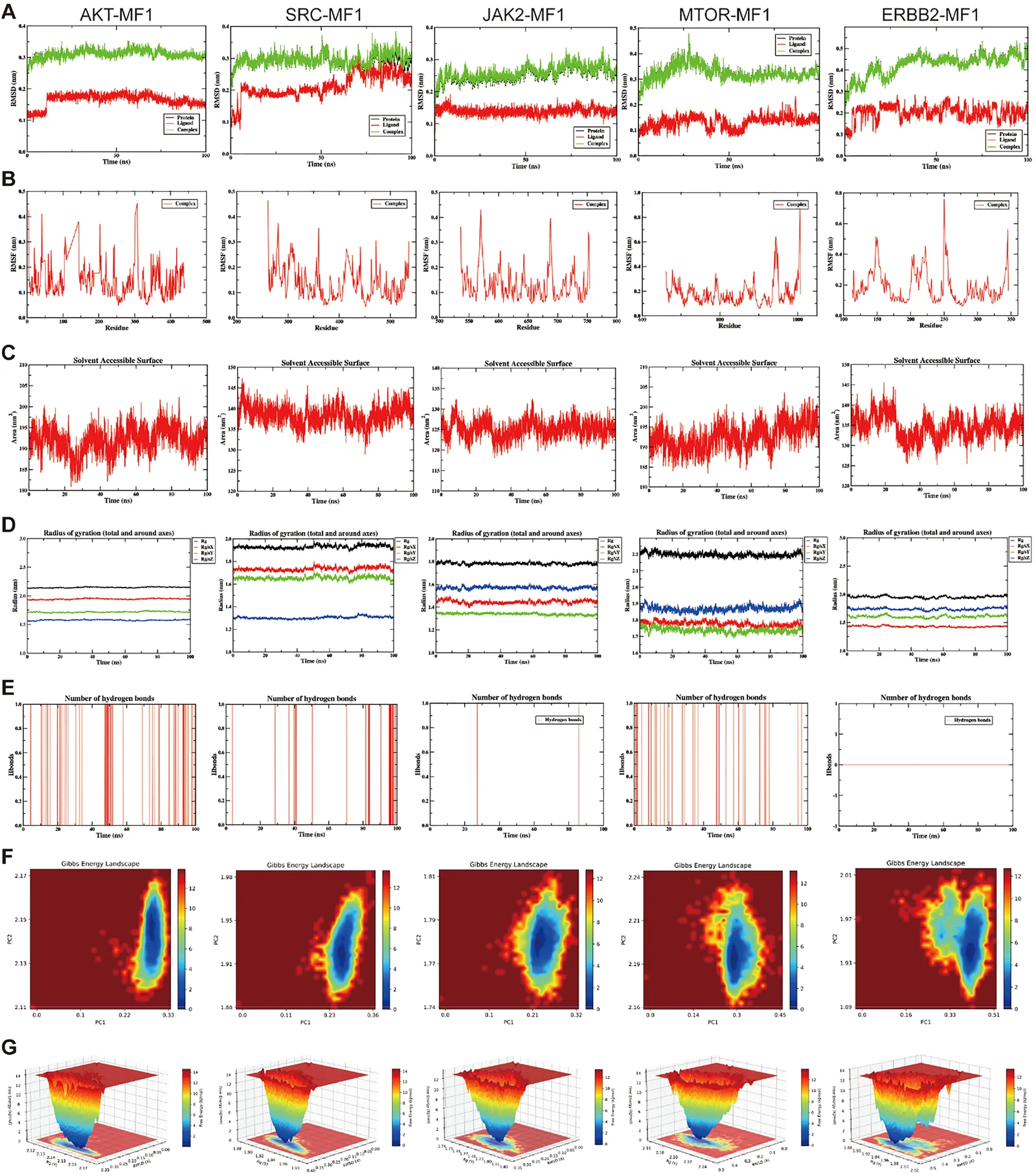
**Molecular dynamics simulations validate the stability of MF1-target protein complexes. A.** RMSD of the AKT1-MF1, SRC-MF1, JAK2-MF1, mTOR-MF1, and ERBB2-MF1 complexes during 100 ns all-atom molecular dynamics simulations. ligand (red) and complexes (green). **B.** RMSF of individual amino acid residues for each target protein in complex with MF1. **C.** SASA of the five MF1-protein complexes over the 100 ns. **D.** Rg of the five MF1-protein complexes over the 100 ns simulation trajectory. **E.** Number of hydrogen bonds formed between MF1 and each target protein during the simulation. **F.** 2D free energy landscape of the AKT1-MF1 complex. **G.** 3D free energy landscape of the AKT1-MF1 complex.

### Single-cell datasets reveal the expression of MF1 targets AKT1/SRC/mTOR as core regulators of immunosuppressive TIME

To validate the association between the core targets of MF1 and the TIME in HCC, we analyzed the single-cell RNA sequencing dataset (GSE166635). Through unsupervised clustering and cell type annotation, we identified 12 major cell lineages, including malignant tumor cells, CD8^+^ T cells, regulatory T cells (Tregs), M1/M2 macrophages, dendritic cells, and stromal cells (Fig. 6A). Density plots and scatter plot analyses revealed that AKT1, SRC, and mTOR were highly expressed primarily in malignant epithelial cells (Figs. 6B–E). This tumor-specific expression confirms that MF1 preferentially targets oncogenic pathways in HCC cells. We further investigated the association between AKT1/SRC/mTOR expression and immune cell infiltration patterns (Figs. 6F). Expression of all three core targets was significantly positively correlated with the infiltration of immunosuppressive cells, including M2-type tumor-associated macrophages (TAMs) and regulatory T cells (Tregs). Conversely, they were significantly negatively correlated with the infiltration of cytotoxic immune cells (including CD8^+^ T cells and natural killer (NK) cells). These findings evidence that AKT1/SRC/MTOR may activation drives the formation of an immunosuppressive TIME in HCC.

**Fig. 6:**
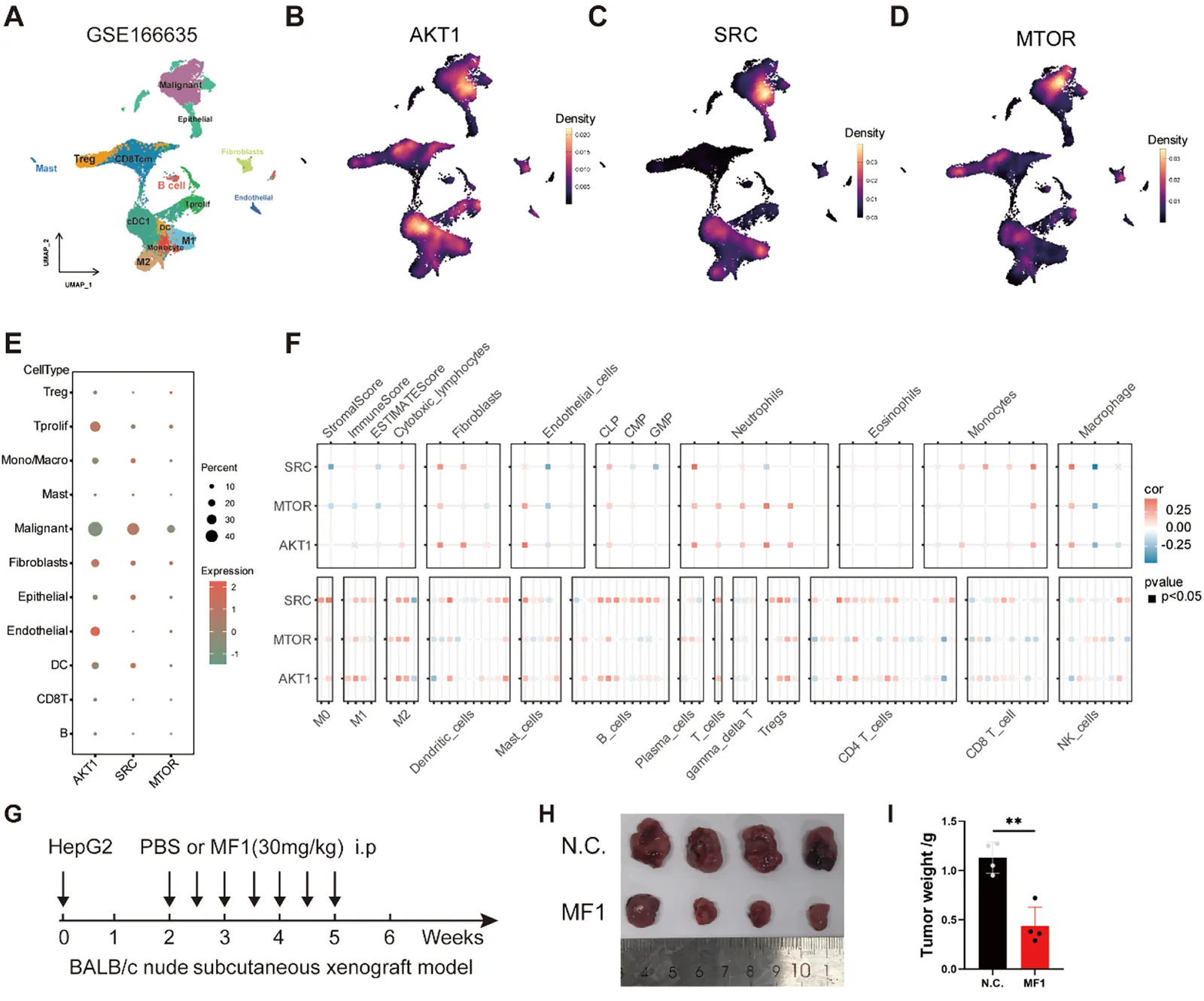
**scRNA dataset analysis AKT1/SRC/MTOR as core regulators of immunosuppressive TIME in HCC. A.** UMAP visualization of single-cell RNA-seq data from the HCC GSE166635.**B-D.** Density plots showing the spatial expression distribution of AKT1 (B), SRC (C), and MTOR (D) across all single cells. **E.** Dot plot illustrating the expression levels of AKT1, SRC, and MTOR in each cell type. Dot size indicates the percentage of cells expressing the gene; color intensity represents the average expression level. **F.** Correlation heatmap between AKT1/SRC/mTOR expression and immune cell infiltration signatures. correlation with stromal score, immune score, and ESTIMATE score and correlation with specific immune cell subsets. Red = positive correlation, blue = negative correlation. Black squares denote statistically significant correlations. *P*-values shown are FDR-adjusted using the Benjamini-Hochberg method, adjusted *P* < 0.05. **G.** Schematic diagram of the HepG2 subcutaneous xenograft model construction and MF1 administration regimen (n = 4). **H.** Representative images of tumors after MF1 treatment. **I.** quantitative analysis of tumor weight in the N.C. and MF1-treated groups. Data are shown as means ± s.e.m. of three independent experiments. Significant difference between the groups, *P < 0.05, **P < 0.01.

### MF1 exhibits significant antitumor activity in vivo

To evaluate the in vivo antitumor efficacy of MF1, we established a HepG2 subcutaneous xenograft model in BALB/c nude mice. Mice were randomly divided into two groups (n = 4 per group) and treated with either PBS (control) or MF1 (30 mg/kg, intraperitoneal injection) for 3 weeks (Fig. 6G). MF1 treatment significantly inhibited tumor growth compared to the control group (Fig. 6I-J). Collectively, these results demonstrate that MF1 exerts its antitumor effects by directly targeting core oncogenic drivers in HCC, which are the key mediators of crosstalk between the TIME landscape, autophagy and ferroptosis.

## Discussion

HCC is one of the most prevalent and lethal malignancies worldwide, with clinical outcomes severely limited by extensive genomic heterogeneity, an immunosuppressive TIME, and resistance to conventional therapies^[^[28], [29], [30], [31]^]^. Most HCC cases develop in the context of chronic liver disease, including viral hepatitis, alcoholic liver disease, and non-alcoholic steatohepatitis (NASH), which are characterized by chronic inflammation, oxidative stress, and fibrosis^[^32^]^. In this study, we demonstrate that the novel RA derivative MF1 exerts potent antitumor activity in HCC cells by simultaneously targeting oncogenic (AKT/SRC/mTOR) and regulating the crosstalk of autophagy and ferroptosis.

A primary challenge in HCC therapy is the immense heterogeneity of TIME landscapes, which makes it difficult to predict patient response to therapies and limits the efficacy of single-target agents^[^33^,^^34^^]^. In HCC cells, sustained AKT/mTOR activation driven by PTEN loss, PIK3CA mutations, hypoxia, or growth factor signaling reshapes the secretion of chemokines, cytokines, and immune checkpoint molecules through downstream effector molecules such as HIF-1α, NF-κB, STAT3, c-Myc, and S6K1. This reprogramming indirectly promotes the recruitment and differentiation of immunosuppressive cell populations while suppressing effector immune cells^[^35^]^. Studies have shown that AKT/mTOR activation upregulates CCL2, CXCL12, CXCL8, CXCL5, CCL22, and CSF1, thereby recruiting monocytes, TAMs, myeloid-derived suppressor cells (MDSCs), and Tregs to the HCC microenvironment^[^36^]^. Our study identifies that MF1 targets 7 core oncogenes (ABL1, ERBB2, PIK3CA, SRC, ESR1, mTOR, and AKT1) in HCC. Notably, the PI3K/AKT/mTOR pathway, the top target of MF1, is aberrantly activated in approximately 50% of HCC cases^[^37^]^. Cai et al. demonstrated that Liuwei Dihuang Pills inhibit the progression of HCC via the PI3K/AKT/TP53 pathway^[^38^]^. In HCC, public cohorts such as TCGA-LIHC and GEO datasets provide valuable resources for validating key targets, pathways, and molecular interactions identified by docking, molecular dynamics, and transcriptomic analyses^[^39^]^. MD results indicate that MF1 forms stable complexes with five proteins, especially AKT and MTOR. AKT and MTOR was significantly positively correlated with the TAMs and Tregs. These may drive the formation of an immunosuppressive TIME in HCC. MF1 may also modulate inflammatory and fibrotic signaling pathways in the HCC microenvironment. The AKT/mTOR pathway is a key regulator of NF-κB-mediated inflammatory responses^[^40^]^, and SRC activation contributes to hepatic stellate cell activation and liver fibrosis^[^41^]^. Patients of HCC may be more likely to respond to MF1 treatment, enabling biomarker-driven precision therapy. Our study demonstrates that MF1 regulates the crosstalk of autophagy and ferroptosis. We observed an increase in ROS and MDA levels, along with changes in GSH and iron ion ratios, indicating that MF1 induces a state of oxidative stress typical of ferroptosis. This finding is consistent with previous research that emphasizes the role of ROS in promoting ferroptosis, as excessive lipid peroxidation can lead to cellular damage and death^[^42^]^. Additionally, we noted a downregulation of ferroptosis inhibitory proteins such as GPX4 and SLC7A11, alongside an upregulation of TFRC, which highlights how MF1 enhances iron accumulation and lipid peroxidation, ultimately facilitating ferroptotic cell death. MF1 also stimulates autophagy, as demonstrated by the presence of autophagosomes and the increased levels of autophagy-related proteins like Beclin-1 and ATG5. Emerging evidence suggests that autophagy and ferroptosis are not necessarily independent; they can be coordinated through mitochondrial damage, glutathione depletion, lipid peroxidation, and iron accumulation, thereby promoting ferroptosis mediated cell death^[^43^,^^44^^]^. MF1 simultaneously activates both ferroptosis and cytotoxic autophagy, providing a dual mechanism to enhance the immunogenicity of HCC cells. In addition, this study has some limitations that need further exploration. The antitumor activity of MF1 was evaluated in three HCC cell lines, and no normal hepatocyte model was included. The MD results provide preliminary evidence of structural stability, but MM/PBSA free-energy calculations and residue-wise decomposition are required to quantitatively assess binding affinity and hotspot residues. Computational predicted AKT1, SRC, and MTOR as potential core targets of MF1. However, direct protein-level regulation and the functional relevance of this predicted signaling axis remain to be experimentally validated. Genetic inhibition of GPX4, ATG5, or Beclin-1 would provide more definitive evidence. In future studies, we will use siRNA, shRNA, or CRISPR/Cas9 to knock down or knock out these genes, validate the efficiency by qPCR and Western blotting, and then assess cell viability, lipid ROS, MDA, Fe²⁺, LC3-II/I, and p62 levels. Bi et al. findings support the value of linking immune-related gene signatures to immune infiltration^[^45^]^. Future studies combine multiplex cytokine/chemokine profiling, co-culture experiments, flow cytometry and chemotaxis assays to determine whether MF1 alters the secretion of IL-6, IL-10, TGF-β, VEGF, CCL2, CXCL8, CXCL12, and CSF1, and whether these changes affect TAM polarization, MDSC expansion, Treg frequency, CD8⁺ T cell exhaustion, and NK cell function.

In conclusion, our study demonstrates that MF1, a novel robustic acid derivative, effectively induces HCC cell death via concurrent activation of ferroptosis and autophagy. Network pharmacology, molecular docking, molecular dynamics, and bioinformatic analyses predicted AKT1, SRC, and MTOR as potential core oncogenic targets and suggested a possible association between cell death pathways and the immunosuppressive TIME. These results highlight the potential of MF1 as a new therapeutic agent for HCC, bridging bench research to bedside clinical translation for patients with solid tumors.

## Ethics approval and consent to participate

All animal-related procedure conformed to the U.S. Public Health Service Policy on Humane Care and Use of Laboratory Animals and were reviewed and approved by the Ethics Committee on Use and Care of Animals of Guangxi University of Chinese Medicine.

## Data availability statement

The data provided in this study are stored in ScienceDB. The data can be obtained by sending an email to the corresponding author. The data link: https://www.scidb.cn/s/AZVnia

## Funding

This study was funded by Natural Science Foundation of Guangxi (No. 2023GXNSFBA026230), Project for Enhancing Young and Middle-aged Teacher's Research Basis Ability in Colleges of Guangxi (No. 2022KY1672), and Research Project of Faculty of Chinese Medicine Science Guangxi University of Chinese Medicine (No. 2022MS007).

## Consent for publication

Not applicable.

## CRediT authorship contribution statement

**Juanjuan Huang:** Writing – review & editing, Writing – original draft, Visualization, Investigation, Funding acquisition, Formal analysis, Data curation. **Lijuan Teng:** Validation, Software, Resources, Investigation, Conceptualization. **Tianxiu Meng:** Methodology, Formal analysis, Data curation. **Yuqiong Liang:** Visualization, Supervision, Resources, Investigation. **Yaoqi Li:** Writing – review & editing, Software, Methodology, Conceptualization. **Jinyan Lv:** Writing – review & editing, Visualization, Supervision, Resources, Funding acquisition.

## Declaration of competing interest

The authors declare that they have no competing interests.
