# Supplementary material for: MF1, a novel robustic acid derivative, exerts antitumor effects in hepatocellular carcinoma
Source: Transl Oncol. 2026 Oct 26;74:103086. doi: 10.1016/j.tranon.2026.103086 (PMC13722059; doi:10.1016/j.tranon.2026.103086)

Figure2F

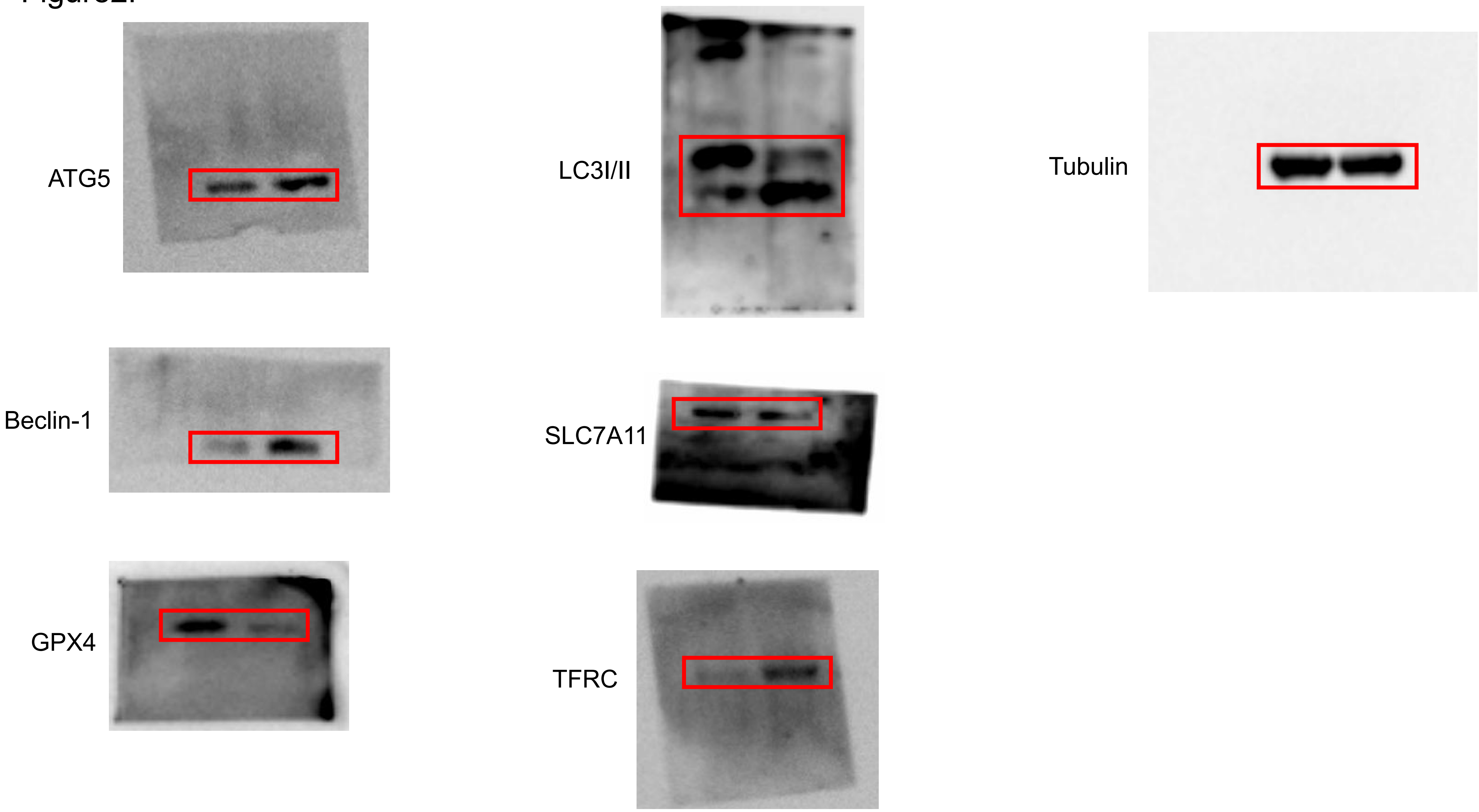

Figure3H

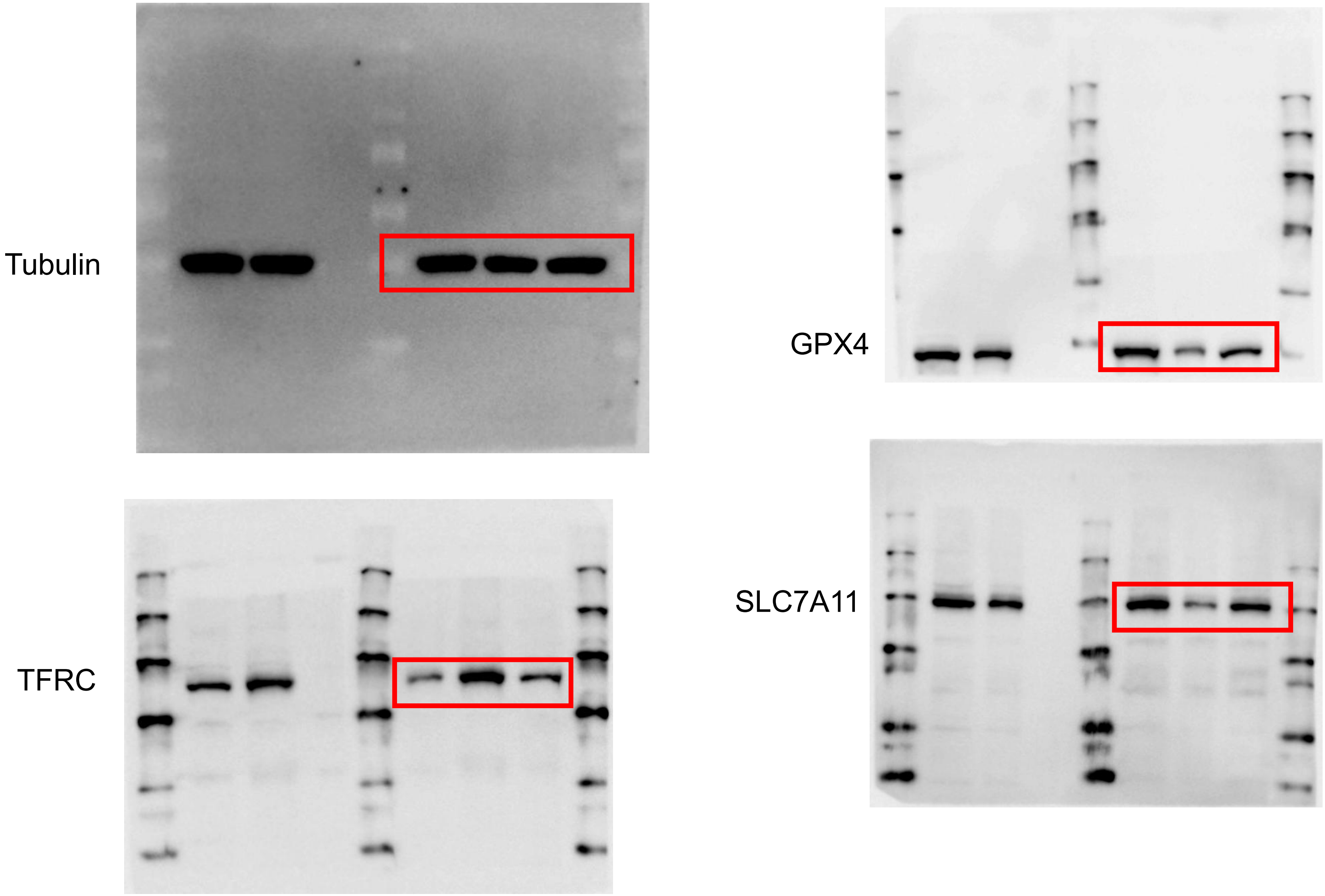

Figure3I

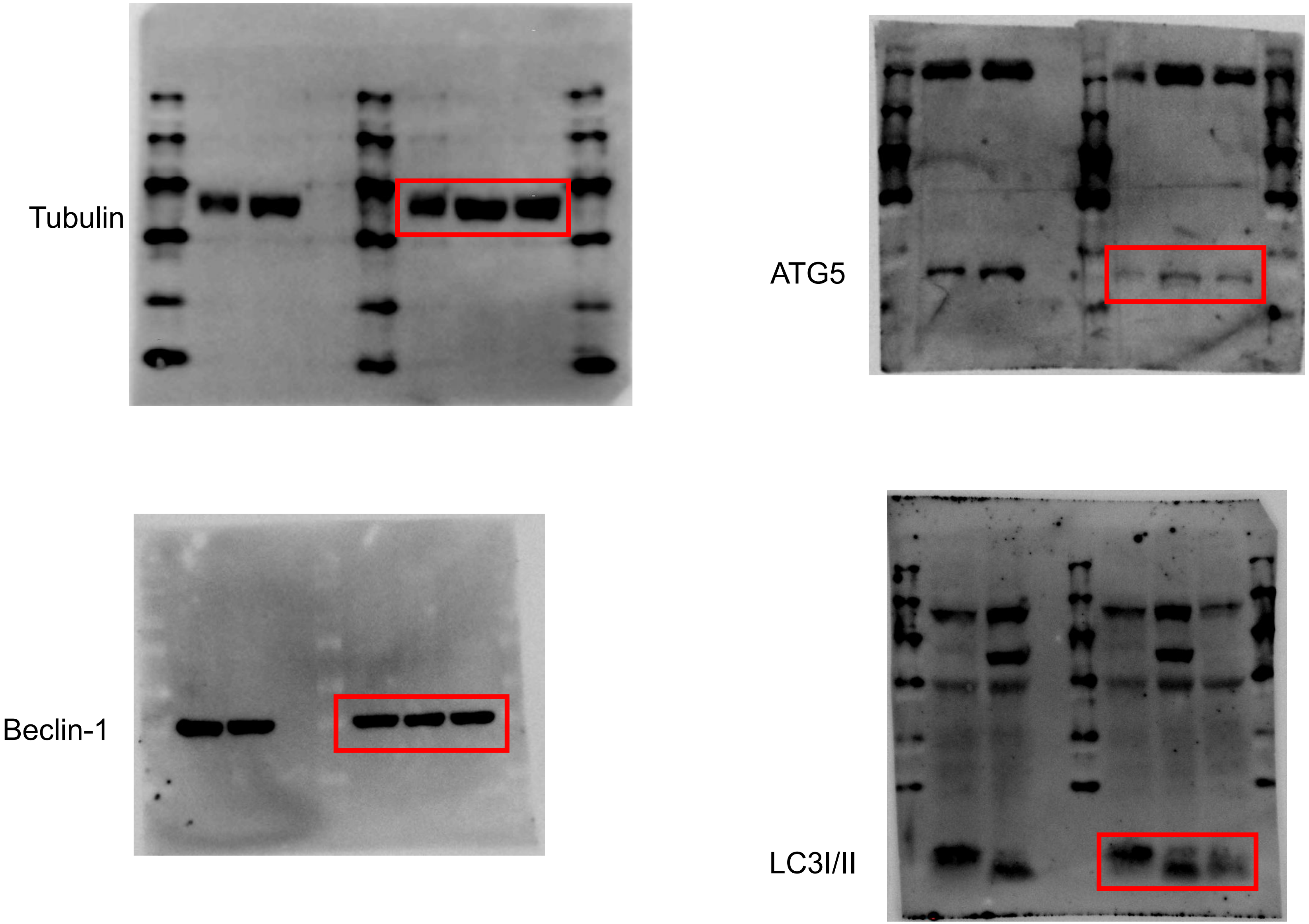

Supplement: Supplementary file 1 [file mmc1.pdf]
